# Sustainable Food Support during an Ultra-Endurance and Mindfulness Event: A Case Study in Spain

**DOI:** 10.3390/ijerph182412991

**Published:** 2021-12-09

**Authors:** Guadalupe Garrido-Pastor, Francisco Manuel San Cristóbal Díaz, Nieves Fernández-López, Amelia Ferro-Sánchez, Manuel Sillero-Quintana

**Affiliations:** 1 Department of Health and Human Performance, Faculty of Physical Activity and Sport Sciences, Universidad Politécnica de Madrid, 28040 Madrid, Spain; fmscd1986@gmail.com; 2 Department of Sports, Faculty of Physical Activity and Sport Sciences, Universidad Politécnica de Madrid, 28040 Madrid, Spain; nievesflopez1@gmail.com (N.F.-L.); amelia.ferro@upm.es (A.F.-S.); manuel.sillero@upm.es (M.S.-Q.)

**Keywords:** sports mindful nutrition, ultra-endurance sports, sustainable foods, antioxidant vitamins, polyphenols

## Abstract

The present industrial food-production system is not suitably ecological for the environment. Mindful nutrition in sport is a relevant emergent sub-discipline that could help reduce environmental degradation. This case study describes a sustainable support diet during an ultra-endurance running (UR) event called the “Indoor Everest Challenge”. This UR challenge involved attaining the altitude of Mount Everest (8849 m) in a simulated way, in less than 24 h, without using ultra-processed food and without wasting plastics. During this challenge, a male athlete (34 years, weight: 78 kg, and height: 173 cm) wore a SenseWear Armband^®^ (BodyMedia Inc., Pittsburg, PA, USA) accelerometer on his right arm to estimate energy expenditure. To supply his nutritional requirements, the athlete consumed only specially prepared homemade and organic food. All consumption was weighed and recorded in real-time; we determined nutrients using two databases: a food composition software, Dial Alce Ingenieria^®^ (Madrid, Spain), to measure energy and macro- and micro-nutrients, and Phenol Explorer Database^®^ (INRA Institut National de Recherche pour l’Alimentation, Paris, France) precisely to determine polyphenolic content. Most energy intake (up to 96%) came from plant foods. We found that subject consumed 15.8 g/kg^−1^/d^−1^ or 1242 g of carbohydrates (CHO), (2.4 g/kg^−1^/d^−1^) or 190 g of proteins (P), and 10,692 mL of fluid. The total energy intake (7580 kcal) showed a distribution of 65% CHO, 10% P, and 25% lipids (L). Furthermore, this sustainable diet lead to a high antioxidant intake, specifically vitamin C (1079 mg), vitamin E (57 mg), and total polyphenols (1910 mg). This sustainable approach was suitable for meeting energy, CHO, and P recommendations for UR. Physical and mental training (mindfulness) were integrated from the specific preliminary phase to the day of the challenge. The athlete completed this challenge in 18 h with a low environmental impact. This sports event had an educational component, as it awakened curiosity towards food sustainability.

## 1. Introduction

As we face the planet’s environmental crisis, humanity must learn to consume more responsibly and conscientiously. In 2009, Rockström introduced the concepts of planetary boundaries (PBs) [1]. Their research quantification suggests that at least four have already been exceeded or are in a zone uncertain, i.e., with high or increasing risk: climate change, land-system change, biogeological flows, and biosphere integrity [2]. The PBs are [2] “intended to represent Earth system processes, which, if crossed, could generate unacceptable environmental change potentially endangering human existence”. Food is one of the human activities with the highest degree of environmental impact, including the emission of greenhouse gases (GhGs). When considering all the activities related to food production, processing, distribution, and consumption it is estimated that 27% of anthropogenic emissions of GhGs worldwide come from the food chain [3]. Nowadays, nutrition habits are changing, the increasing massive consumption of animal foods has been identified as a significant determinant of unsustainability [3]; meat production accounts for one of the most critical adverse environmental impacts on the Earth. Specifically in Spain, from 1960 to the present, the livestock sector has shown enormous development [3]. GhGs emissions from livestock production since the beginning of the century in Spain have increased from 8 to 75 million tons of CO_2_e per capita, per year. It has also been estimated that most (81%) of the total GHG emissions derived from the food chain from the Spanish population’s production are associated with food of animal origin [3]. Another nutritional habit change is the massive consumption of over-packaged ultra-processed foods (UPFs) [4,5], which is also questioned from the sustainability point of view. Significant improvement in the food system’s sustainability requires urgently encouraging limiting UPFs to the benefit of mildly processed foods, preferably seasonal, organic, and local products [6] and reducing plastic waste. In 2019 plastic demand by segment for packaging (39.6%) represent the larger end-user market. Within over-packaged foods are the UPFs associated with imbalanced nutritional intake [4,5,7]. Ultra-endurance events and participation numbers have increased progressively over the past three decades [8]. Endurance events produce environmental damage with enormous plastic bottles waste and plastic packaging from gels, bars, and over-packaged ultra-processed foods. Several actions to reduce plastics waste in sports races, such as the London Marathon Event, a world leader in sustainable mass participation events, had proposed reducing the drink stations from 26 to 19; this could remove around 215,000 plastic bottles [9]. In addition, the last Xiamen Marathon, set to use no plastic, used the message “save the ocean” to show the importance of plastic waste and its environmental impact. In 2019, China contributed 31% of the world’s plastic production, an enormous level considering that all of Europe (EU27, UK, and NO/CH) represents 16% of the world’s plastic production. Evidence shows a trend to a reduction in Europe between 2018 and 2019 [10]. Experts agree that balanced hydration and nutrition play a crucial role in athletes’ health and optimize performance [10,11,12]. We must change how we cope with these energy and fluid demands using compostable cups, introducing drop zones across the course, or using reusable cups [6]. Athletes competing in ultra-endurance running (UR) activities need an adequate intake of energy, carbohydrates (CHO), proteins (P), and antioxidants during training and competition. Optimal nutrition intake is relevant to ensure optimal performance and recovery and minimize health risks. UR athletes have described a high prevalence of gastrointestinal (GI) distress [11,12,13], which could increase the difficulty of meeting nutritional recommendations during UR running [8]. The pathophysiology of GI distress in ultra-runners is not fully understood, and it is likely multifactorial. However, an appropriate fluid, CHO, P, and fat intake during the UR appear to be essential in developing GI symptoms [11,13]. In this way, ultra-endurance runners without GI distress had a fluid and fat consumption rate almost double that of runners with GI symptoms [11].

Most studies demonstrate improved endurance performance when subjects consume different amount and types of CHO [8,14]; specifically, a benefit was found when CHO intake (as liquid) was compared with water [15]. The position on fluid intake [16] highlights the need for an individualized drinking pace according to sweat rates. In a specific preparation phase, two different positions [12,16] were recommend, encouraging athletes to slowly increase CHO intakes to at least reach the threshold of 80 g/h. A field study [17] showed a significant positive correlation between CHO intake and faster finishing times in Iron Man and marathon races in a large (*n* = 221) population of athletes.

Participants in sport endurance events, such as ultra-endurance running, are recommended to consume a high daily carbohydrate (CHO) intake of up to 12 g/kg/d [14]. This level of CHO is complicated to reach, and there must be a previous organization of meal patterns [8,12,18,19]. Endurance athletes often include sports supplements intake [12,14,19,20,21] to reach high daily nutrient recommendations, specifically in endurance sports, to cope with this high CHO requirement. Training distances of ultra-endurance runs are associated with a high level of mechanical stress [12] that could be related with muscle damage, inflammation, or soreness. A wide assortment of nutritional supplementation strategies has been investigated to reduce the consequences of this physical stress [22].

Among the micronutrients, we must highlight the antioxidants that constitute a heterogeneous group of substances of very different chemical natures, such as polyphenols (PPs)—which are common antioxidants present in many foods and beverages of plant origin [23,24]—with the most popularly researched being quercetin, catechins, and resveratrol [23]. The sports nutrition guidelines [14,16] do not include the requirement for this type of antioxidant. This group of substances—polyphenols—are characterized by their structure of one or several phenolic groups, capable of reducing reactive oxygen species (ROS) [15]. During endurance running, mitochondria’s increase in oxygen consumption would augment ROS formation at the electrical transport chain. During physical training, oxidative stress is defined as a state of cellular imbalance between the production and the capacity to neutralize ROS [15]. It has been referred [25] that polyphenols intake is related to health benefits; nevertheless, more research is needed to identify them. PPs lead to an improvement of vasodilator, antioxidant, and anti-inflammatory properties, which determines an enhancement in blood flow, and a reduction in oxidative stress and muscle inflammation related to a benefit in physical performance, specifically during endurance activities and mainly associated with reduced muscle damage [22].

In this same sense, from damage to recovery, the planetary damage requires a solution that leads us towards developing consciousness of care for the Earth to accelerate its recovery through a change in eating habits to reduce the environmental impact.

According to Meyer et al. [26], “A sustainable diet has a low environmental impact, contributing to food and nutrition security and a healthy life for present and future generations”. Meyer et al. [26] define the five steps to a sustainable diet for athletes.

Although it is well established that protein intake recommendation is higher for athletes [14], one step to a sustainable diet for sports [26] recommend reducing the overall consumption of proteins, specifically meat, dairy, and supplements. In addition, the quality of food from production to consumption needs to be considered for a sustainable diet: fresh, organic, locally produced, and diverse [26]. The 2020 Household Food Consumption Report, published by the Spanish Ministry of Agriculture, Fishing, and Food [27], shows for the first time that ecological product intakes represent 4% of total food expenses, mostly locally grown fruit and vegetables. This report shows a growing trend to produce and consume locally grown products. On the downside, this report shows a 10.9% increase in fresh meat consumption after several years of decline [27]. Nowadays, meat production accounts for the most significant adverse environmental impact on the Earth. However, a Scottish study [28] found a general lack of awareness regarding the link between eating meat and climate change [29].

Meyer et al. [26] proposed that teams and institutions initiate a practical application for a sustainable diet in sports. To meet the nutrition guidelines for performance, sport-nutrition professionals should consider environmental impact when making food recommendations. Some athletes feel they do not need to eat less meat because they have already reduced their consumption. Skepticism of scientific evidence linking meat and climate change is already widespread [29].

On the other hand, educational programs for developing awareness, curiosity, patience, and attentional skills are focused on developing a mindful openness state that could lead our diet behavior to more sustainable food habits, thus changing the default net system that leads us to automatic food intake [30]. An exciting study area is the effect of Mindfulness (MF) training on ecologically responsible consumption behavior through mindfulness-based interventions (MBIs).

The last two decades have seen exponential growth of mindfulness research [30], and different programs derived from the initial curriculum designed by Kabat-Zinn [31], “*Mindfulness-Based Stress Reduction*” (MBSR), have been applied in different frameworks. MF training could promote a sustainable lifestyle and behavior. MBIs are the way to change unhealthy habits, improving self-care. Jon Kabat-Zinn [31] defined MF as “the awareness that emerges through paying attention on purpose, in the present moment, and non-judgmentally, to the unfolding of experience moment by moment”. A specific MF curriculum, “Mindful Climate Action” [30], had been proposed for helping people to improve their health while simultaneously lowering their carbon footprint. This education, Mindful Climate Action, is spans eight weeks and, like MBSR, aims to improve personal health and wellbeing and shift the diet towards plant-based foods, and, at the same time, reduce unnecessary purchasing and consumerism.

Furthermore, a cross-sectional study [32] with a large population (*n* = 310) that included participants with and without meditation experience found that the constructs of MF can play an essential role in building up motivation for a change of behavior toward sustainability. Mindless eating is at the nucleus of many ecological and social problems associated with modern nutrition behavior [33]. An adult student’s population following a MBI was analyzed for food habit changes, and it was concluded that changing habits toward more sustainable food choices is a slower process [33]; however, MF training has been proven to be efficient for improving healthy nutrition habits [33]. It was also suggested that mental training through mindfulness, focused on developing kindness and compassion, leads to openness and curiosity in difficult situations, instead of avoiding or suppressing it, bringing a sense of wellbeing and inner calm [34].

The objective of this case study was to prove that the nutritional needs of even an ultra-endurance athlete can be met with sustainable, organic, locally, seasonally, and unprocessed food without using packaging or plastic waste. Furthermore, extreme athletes can serve as role models to encourage healthier food habits in the general population.

We found a knowledge gap about a holistic approach using mental (MF) and physical endurance to train and empower athletes to follow a more sustainable diet. A limitation of this observational study is that it could be considered a pilot study—a single case—and we know that the unusual physical exercise (indoors, ascending, and descending stairs) is very different from other UR events.

This study aimed to describe the energy, CHO, several antioxidants including polyphenols, and fluid intake from sustainable snack support during a mindful UR event in Spain; and to encourage other athletes to consume a more sustainable diet aligned to the PBs framework of Sustainable Developments Goals, as defined by the United Nations [35].

## 2. Materials and Methods

“Indoor Everest Challenge”:

The experimental design test was an ultra-endurance running event, completed indoors, which, due to the simulation and the vertical distance reached, we named the “Indoor Everest Challenge”. Nevertheless, the environmental conditions were normal, and oxygen availability was not compromised.

The subject completed an ultra-endurance event; he was ascending and descending the 7 floors (=1 cycle) of the Faculty of Physical Activity and Sports Sciences from Saturday 7 a.m. to Sunday at 1 a.m. He completed a total of 313 cycles in 18 h.

The event began on the ground floor. On the 7th floor, we controlled the cycle number completed and offered him sustainable whole food and drink support. On this same floor (7th), each hour the athlete passed a physiological control, and we installed a rest zone for settling down and stretching.

During the duration of the event, each hour, our athlete passed a physiological control for testing different parameters: heart rate, blood glucose, arterial pressure, and oxygen saturation. He was asked about self-perception of exercise (RPE) and pain (RPP) rates.

### 2.1. Participant

The subject case of study was a male, aged 34 years, with a body weight of 78 kg, a height of 173 cm, and a body mass index of 25.7 kg/m^2^.

This participant did not have a previous background in endurance training; nevertheless, his physical condition was optimal due to prolonged (more than ten years) and systematic (2–3 h/day) physical training; practicing CrossFit, weightlifting, and general calisthenic exercises. Informed consent was obtained, and the event was developed according to Helsinki Declaration.

A preliminary anthropometric study classified him as mesomorphic with a high lean body mass (51.9%) and a low body fat (6.4%).

Approximately three months before the race day, he decided to follow specific endurance training for this challenge precisely, he ascended and descended stairs for 1–2 h per day, running 8 km daily, and 20 km once weekly.

### 2.2. Specific Preparation Phase

This study was observational. During regular daily physical training, the oxygen intake at a paced rhythm (21 cycles/h) was measured in a preliminary stage. Furthermore, we used the weighted method (weight measurement before and after the training) to calculate the sweat rate and individualize the needed fluid intake. These data were measured to estimate energy expenditure (~8000 kcal) and the sweat rate (~600 mL/h) at the same speed (21 cycles/h). We previously estimated the amount of food and drinks offered during the event day.

The athlete tested different homemade sports drinks and snack bars in this preliminary phase, varying their ingredients to higher or lower CHO and P concentrations. Every food and drink item chosen for the challenge was tasted for several weeks in this preliminary period.

### 2.3. Mental Training

As part of physical training, mental training was practiced—precisely, formal meditation, to develop self-acceptance. We chose several mindfulness exercises from two validated mindfulness protocols (MBSR and CCT), explicitly focused on kindness, self-compassion, meditation, and different breathing exercises. A mindfulness program—8 weeks long—focused on body consciousness and connection, was followed daily in the previous months. The last meditation, 24 h before the event, entitled “Self-compassion”, lasted 30 min. During the event, the athlete practiced several breath exercises focusing on present moment awareness.

### 2.4. Food Intake

The nutritional intake was actively monitored during the event. All food (Table 1) and drink (Table 2) was consumed exclusively from the total support snacks offered during the event.

Every food and drink item intake had been weighed using an electronic scale (Mettler-Toledo SAE^®^, Barcelona, Spain) to 1 g accuracy. All nutritional data were analyzed using the Dial^®^ version 2 software (Alce Ingenieria, Madrid, Spain), and every home recipe was introduced. To determine the food’s different phenolic contents, we used the Phenol-Explorer version 3.6 [36].

Due to the tremendous oxidative stress associated with ultra-endurance events, we have focused on selecting fruits, seeds, and spices with a high concentration in antioxidant micronutrients that have previously been described as the best sources [24].

An ecological support snack regime, based on non-ultra-processed foods, according to NOVA food classification [5], mainly classified unprocessed or minimally processed from diverse plant sources, was carried out. All food and drink consumed was homemade without plastic packaging and followed a sustainable nutrition approach from organic agriculture products.

Furthermore, we used glass bottles, which were straightforward to clean and fill during the event and suitable for all the drinks.

Every beverage was prepared in real-time according to the athlete’s demands, except the broth, which was previously cooked with fresh vegetables and ecological farm poultry.

## 3. Results

### 3.1. Energy, Carbohydrates, Proteins, and Lipids

**Figure 1:**
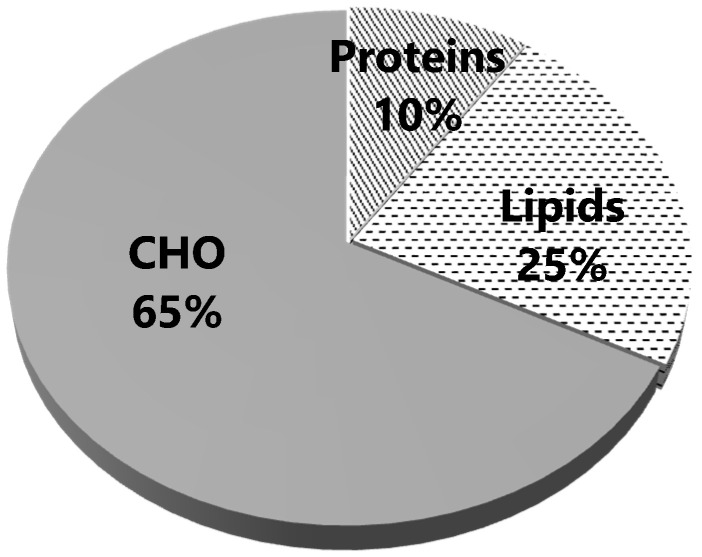
Energy intake and its distribution from the three macronutrients: proteins, lipids, and carbohydrates (CHO).

The subject consumed 15.8 g/kg^−1^/d^−1^ (1242 g) of CHO, 190 g of P (2.4 g/kg^−1^/d^−1^), and 207 g of L. The total energy intake (TEI) was 7580 kcal; the energy distribution (Figure 1) shows that CHO contributes almost two-thirds to the TEI during the event. A proportion of 10% of TEI came from protein intake (Figure 1).

The energy intake came from six categorized food groups (Table 3). The highest energy was provided from the first food group, represented by fresh fruits (bananas, tangerines, kiwis, and blueberries) ingested in a raw state, and several dry fruits and nuts, which were the ingredients of the snack bar. The grain and seeds group provided more than a quarter of total energy intake, including oat, wheat, and derivates, such as spelt flour and several seeds, all as ingredients of fresh bread and snack bars.

Animal food items (eggs, smoked cod, and poultry) were consumed, respectively, through the snack bars, ready to eat for filling sandwiches, and a homemade broth. The sixth group was mainly represented by extra virgin olive oil, used to cook eggplant to fill sandwiches. Pure dark chocolate was offered mainly during the last phase event.

CHO intake (1242 g) came from items included in three different food groups (Table 4) and represent the more significant proportion of energy intake (Figure 1).

The proteins were derived from four different food groups (Table 5). Although the fifth group—animal foods—was the lowest related to energy intake (Table 3), we observed that ~25% of total protein intake came from the animal food items (eggs, poultry, and cod). However, the smoked cod was the only one directly ingested; the other two food items (eggs and poultry) were part of cooking the energy bars and broth, respectively.

The overall event was divided into 6 phases that lasted 3 h each. The table shows the average intake of CHO, P, and fluid intake per hour. During the event, the average CHO, P, and fluid intake per hour were, respectively, 81.3 ± 21.2 g/h, 10.5 g/h, and 594 mL/h.

A proportion of 25% of TEI came from L, derived from 5 different food groups (Table 6), distributed as saturated fat (42.5 g), monounsaturated fat (76.5 g), and polyunsaturated fat (PUFA) (43.8 g). Ingestion of 867 mg of n-3 PUFA, represented specifically by eicosapentaenoic acid (EPA) and docosahexaenoic acid (DHA), contributed 1.2% of the TEI.

UPFs were not offered, but several foods items, which were classified by NOVA [5] as processed food—smoked cod, fresh bread, boiled eggplant with extra virgin olive oil, and oat snack bar—were provided and considered as mildly processed food. The highest energy was derived from the first group mainly unprocessed or minimally processed, as represented by fresh fruits (bananas, tangerines, kiwis, and blueberries), ingested in the natural state, and some dry fruit (dates and raisins) and nuts (almonds and cashews), which were ingested as ingredients in the oat snack bars. The grain group includes oats, wheat, and derivates, such as spelt as ingredients of the fresh bread and snack bars.

Animal food items (eggs, smoked cod, and poultry) were consumed through the snack bars, ready to eat for filling sandwiches, and cooking homemade broth, respectively. The miscellaneous group was mainly represented by extra virgin olive oil, the main ingredient for cooking eggplant filling sandwiches, and pure dark chocolate.

We divided the whole event into six periods of three hours; Table 7 shows the meal corresponding to each period and the average CHO, P, and fluid intake per hour in each phase.

The average intake related to body weight (g/kg/h) of three macronutrients (CHO, P, and L) (Table 8) shows a more significant intake related to weight per hour from CHO and a similar pace for ingesting protein and lipid during the event.

### 3.2. Fluid Intake

**Figure 2:**
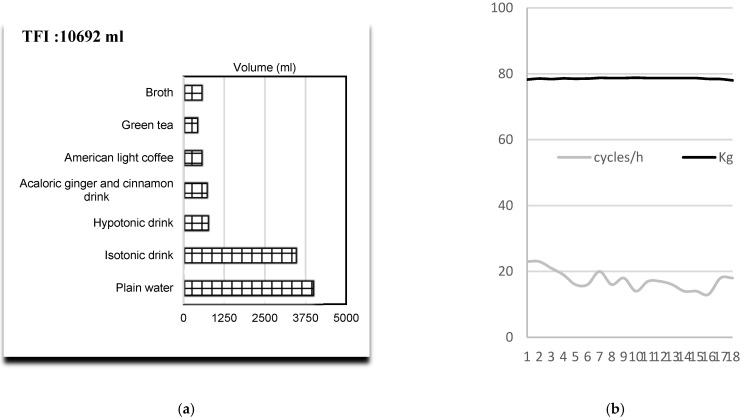
(**a**) Volume intake (mL) and percentage of contribution to the total fluid intake (TFI). (**b**) Bodyweight (kg) and number of cycles completed during the 18 h event.

The figure below (Figure 2a) illustrates the total fluid intake (10,692 mL) provided by 7 different drinks, supporting 22% of TEI (Table 3), including 338 g of CHO. Plain water and isotonic homemade drinks with lemon and honey were the most consumed (Figure 2a). However, warm beverages, such as green tea (464 mL), American coffee (582 mL), and homemade broth summed a total volume of 1651 mL. We observed minimal weight modification: at the end of the event, the lost weight was 250 g (Figure 2b).

An extra water intake (3553 mL) was derived from solid vegetal foods’ water content, mainly in fruits; the percentage of water in several food items consumed varies from 75% in bananas to 94% in tomatoes. In this way, whole bananas (1564 g) and tangerines (1246 g) intake provided more than one liter of water each—1182 mL and 1246 mL, respectively. Other solid foods contributing to water intake were blueberries (406 mL) and tomatoes (296 mL). We found a high intake of pure lemon (688 g); however, this item was included in beverages or drinks intake.

### 3.3. Antioxidants Intake

Vitamin C intake (1079 mg) derived mainly from fruits and the better sources were tangerines, lemon juice, kiwis, and blueberries. Other plant foods, such as eggplant, avocado, and tomatoes were also important vitamin C sources due to their content in dry fruit, muesli classified as a grain, and derivates, which also enhanced vitamin C consumption.

The vitamin E intake (57 mg) derived mainly from almonds (23%), sunflower seed (33%), extra virgin olive oil (11%), and spices or condiments (oregano, cinnamon, and paprika) were also essential providing vitamin E.

**Figure 3:**
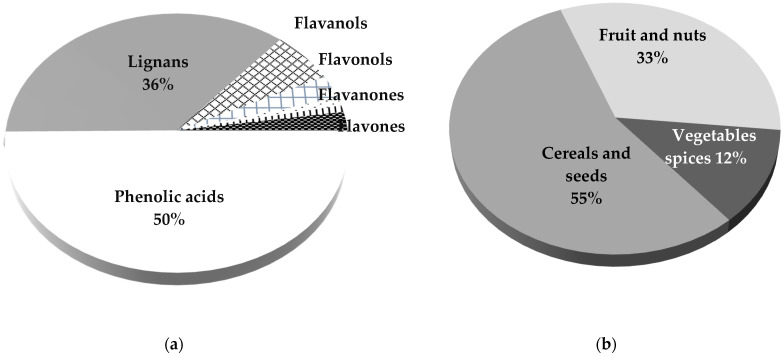
(**a**) Different polyphenols (PPs) intake. (**b**) Antioxidant substances (AS) intake by food groups.

The polyphenols (PPs) group products include more than 500 substances; we have categorized them into 7 substance groups (phenolic acids, lignans, flavanols, flavonols, flavones, flavanones, and others) to express their percentage contribution (Figure 3a). Phenolic acids—mainly garlic and rusmaninic—were provided from bananas, eggplants, and oregano; the flavanols represented by catechins were provided by almonds and bananas; the lignans were derived from tangerines and garlic; the flavanones group, represented mainly by the hesperetin, came from pure lemon juice and tomatoes. The flavonols group, such as quercetins and kaemphenol, derived, respectively, from blueberries and almonds. The total PPs intake (1909 mg) was mainly represented by phenolic acids, followed by the lignans group (Figure 3a).

Related to their content in PPs, seeds were good sources: sesame seed (158 mg), wax seed (125 mg), and sunflower seed (74 mg). Nevertheless, most PPs intake (~44%) came from green tea, coffee, and dark chocolate. Fresh fruits intake (blueberries, lemon juice, and tangerines) was crucial for PPs intake, providing 29% of the total intake. Other spices and herbs consumed in lower quantities, such as cinnamon, garlic, and dry oregano, were crucial.

When we analyzed the antioxidant substances (AS) intake (vitamin C, vitamin E, and PPs) in the total snack support intake, we found that over half (55%) came from grains and seeds (Figure 3b), one-third of AS derived from fruits and nuts, and the rest (11%) derived from different spices, herbs, and condiments.

### 3.4. Physiological Parameters

The rest heart rate was 55 bpm, and during the event, the average heart rate was 145 ± 20 bpm. The SenseWear Armband^®^ accelerometer observed that more than half the total time expended (up to 11 h) was classified at a moderate intensity (Table 9), and a for long time—more than 4 h—the physical load was classified as intense. Resting time, lower than an hour (Table 9), was used for eating and monitoring physiological assessment. Indeed, twice during the race, the athlete sat down in a chair, putting his legs over a cylindric yoga roll, having a short rest while eating at the same time.

Moreover, the speed rate (cycle/h) was maintained between 14 and 23 cycles per hour (Figure 3b). The minimum speed (14 cycles/h) at the 10th hour was concomitant with a high rate of self-pain perception (RPP = 9/10). The total covering distance was 75.81 km, the total steps number was *n* = 95,546, and the average metabolic unit (MET) was 4.8 during the whole event.

## 4. Discussion

The position statement (ACSM, 2016) [14] recommends a CHO intake during ultra-endurance (>2.5–3 h) up to 90 g/h. In our case, the CHO intake of 81 g/h, close to this recommendation, shows that the food and beverages provided during the event, planned for hydration and fuel, were a success. Even until the last period—phase 6 or night snack (NS)—the athlete achieved a CHO intake of 45 g/h.

It has been suggested that ultra-endurance athletes fail to meet nutritional and water recommendations [8,18]. In our case, the athlete met an optimal diet composition (65% CHO, 25% F, and 10% P) and total energy intake over 7580 kcal: he reached 81 g CHO intake per hour. A study [37] in three different mountain endurance events found a similar energy distribution (71% CHO, 21% F, and 6% P), but CHO intake was lower (31 g/h) during the race. Stellingwerff et al. [19] proposed nutrition approaches in elite marathon runners leading to an individual race day fluid and fuel plan. After adaptation to handling intake, increased CHO intake during the race until 61 ± 15 g/h. These elite marathoners consumed 15 g of CHO in 150 mL every 15 min; they used commercial products with high content CHO in the form of sports drinks and gels. In our case, the athlete consumed 20 g of CHO every 15 min without using such sports supplements. Since 2009, the concept of UPFs from NOVA classification was coined for industrial formulations as those products manufactured from substances derived from foods or synthesized from other organic products and those with additives [5]. The UPF term has rapidly emerged and is now recognized and used both by public institutions (e.g., FAO, WHO) and academic researchers worldwide [5,6]. Some standard UPFs products are carbonated soft drinks or energy drinks and sweet, fatty, or salty packages snacks, which are widely used; nevertheless, the participant avoided them and had not reported GI symptoms. However, a study [13] using a post-race questionnaire about dietary intake and gastrointestinal distress found on the 60 km race day that 82.9% of the athletes reported some GI symptoms during the race. A negative correlation for most GI symptoms was found for CHO, energy, and fluid intake; nearly one-quarter of 60 km runners achieved the recommendation to take >60 g of CHO per hour and 14.6% consumed >500 mL/h of fluid. In our case, the average fluid intake was 594 mL/h. Furthermore, as referred to previously [19], a study of elite marathoners (604 ± 156 mL/h) and a small group (*n* = 4) of 120 km runners, observed continuously during the race day [13], showed fluid intake between (392–609 mL/h) and found a significant variation in CHO intake during the race in 4 ultra-endurance athletes that varied between 31 g/h and 108 g/h. In order to clarify the controversies about hydration strategies, they found that drinking to quench thirst was the most common (67%), and recommendations to ingest sodium and to drink to avoid more than 2% body weight loss are not universally supported by the scientific literature [38]. In our athlete, we found a weight variability (+2% −0.8%) during the event and a trend to consume less fuel and fluid in the latest phase (from 15 h to 18 h); nevertheless, he was drinking to quench thirst throughout the event.

It is well known that dietary protein intake is strongly determined by total energy intake [21]. In many sports, recent trends show a higher intake of protein than recommended [14,21,26]. The same behavior is shown for the Spanish population; between 1960 and 2010, animal protein consumption multiplied by 2.6, and more than 75% was derived from animal sources [3]. The athlete provided a higher protein intake (2.4 g·kg^−1^·d^−1^) when considering the ACSM recommended range (1.2–2 g·kg^−1^·d^−1^); furthermore, the total energy intake (TEI = 7580 kcal) was intensely high and most proteins consumed originated from plants (75%). In another study [21] of a group of marathon runners, plant-based protein provided was lower (40%); while Beis et al. [39] found, in elite Kenyan endurance runners, that 76% of the protein came from plant protein and 24% from animal sources. Both studies [21,39] reached the protein recommendation (1.8 g·kg^−1^·d^−1^), suggesting that protein supplementation is not required to meet the current proposal for daily protein intake. Plant proteins are crucial as protein sources and represent a better choice for planet sustainability. Furthermore, emerging evidence suggests that the food matrix consumed protein may directly influence the post-exercise muscle protein synthetic response in healthy young adults [40].

The lowest impact animal product typically exceeds vegetable substitutes [41]. The importance of dietary changes in selecting food toward more sustainable nutritional habits is exceptionally complex and is affected by the whole food chain. Farms, processors, and retailers have suggested an integrated mitigation framework, and consumers favor sustainable consumption [41]. Sales data from a representative sample of food retailers in Norway showed a 48.8% of food expenditure in 2013 was derived from ultra-processed food [7]. In Spain, the annual report on food consumption of Spanish households in 2020 [27] shows an increase in the consumption of packaged food and a reduction in bulk purchases. Between 1960 and 2010, in Spain, the stages of the food chain after food production—processing, distribution, and consumption—increased from 18 to 43% in GhGs emissions [3]; part of this increase could be related to plastic waste, releasing GhGs emissions during an unknown time, mainly in the form of ethylene and methane [42]. Indeed, in Spain, the percentage of UPFs purchased almost tripled between 1990 and 2010 (from 11% to 31.7%), of which, more than 80% were ultra-processed foods with added sugar [4]. The increased food processing technologies and the use of derivative protein products (whey, casein, soy, and pea protein supplements) to meet daily protein requirements have gained widespread popularity among physically active individuals due to convenience [20].

A lack of congruence and awareness among people using supplements has developed curiosity research over the behavior of supplement users [43]. In this way, over half (54%) of 872 users reported experiencing side effects associated with this type of multi-ingredient pre-workout supplement [43].

Nowadays, it is crucial to propose more information on new planetary health plates reducing the meat content and the sports supplements abuse to cope with nutritional demands in different sports. More balanced food consumption and production approaches are needed: reduced meat and dairy are crucial to reducing GhGs [2].

On the other hand, a sustainable snack-based diet regime, mainly from plant sources, could reduce oxidative stress due to its high antioxidant properties, including polyphenols, vitamin C, and vitamin E. A list of the 100 richest dietary sources of PPs [24] shows that the highest number of items in this list group is the seasoning groups, followed by fruit and seeds. Concerning our result, a dozen food and beverage intakes were included in this richest list of dietary food—sorted according to their concentration from highest to lowest, these were: oregano, dark chocolate, blueberries, dried ginger, almonds, green tea, whole grain wheat flour, extra-virgin oil, pure lemon juice, whole grain oat flour, walnut, and vinegar.

The total PPs content was calculated as the sum of all individual PPs and a list of 89 foods and beverages providing more than 1 mg of total PPs per serving. We checked out this second list (89 food and beverages), providing around 1 mg PPs per serving size, and we found ten foods or beverages items intake; specifically, blueberries, flaxseeds, dark chocolate, green tea, almond, whole grain wheat flour, extra virgin olive oil, pure lemon juice, bananas, and tomatoes. As we can observe, some spices, such as dry oregano, do not appear in this second list; this dry herb is usually consumed in small amounts, so the serving size is very small. However, in our case, the oregano and the garlic, not included in this 89 food and beverage list, were added to the recipes in higher amounts than usual, and both were essential sources of PPs and antioxidants vitamins during this event.

Indeed, PPs show highly diverse structures, over 500 different molecules are known in food [24]. Our data found that blueberries provided many flavonol groups substances, specifically different types of quercetin, and dried oregano was an excellent source of phenolic acids, such as Rosmarinus acid. However, we probably have sub-estimated the total amount of PPs due to several data having been missed; some food items (e.g., hibiscus, leeks, chilli, and poppy seeds) were not found in the Phenol Explorer Database, even if it is, today, the most complete database on the content of PPs in food, including 452 foods and beverages [36].

A review analyzing the protective effect of PPs in exercise [22] shows different plant food (fruit, herbs, and seeds) rich in antioxidants that could reduce muscular damage or soreness, leading to a better muscular response to physical stress. Nutrition data from both software (Phenol Explorer Database for PPs and Dial Alce for vitamins) showed that different food items consumed by our subject have PPs and vitamins C or E, such as tangerines, almonds, and pure lemon juice. Tangerines were excellent sources for lignans and vitamin C, the almonds have a high amount of vitamin E and catechins, and the pure lemon juice has a high content in hesperetin (flavanone) and vitamin C. Indeed, these databases could have sub-estimated the amount of the micronutrient in the foods because the databases of food composition did not consider the different agriculture methods (conventional versus organic) for growing these plants. However, [44] suggests a higher content in micronutrients (5.7%) in organic plants when compared with those grown under conventional agriculture methods. All plant foods ingested were organic, and data corrected for vitamin C and vitamin E will be, respectively, approximately tenfold the dietary reference intakes (DRIs) [45], without using supplementation, only through intake of whole organic foods. Furthermore, a meta-analysis found [46] a higher antioxidant amount specifically more evident for the phenolic groups of flavones and flavonols that were highest in organic plants. They concluded that organic crops have higher antioxidants content, lower concentrations of cadmium, a toxic metal, and a lower incidence of pesticide residues than the non-organic comparators across regions and production seasons [46]. Well-regulated organic products must be a primary option for following a sustainable and healthy diet. Nowadays, mindfulness can play an essential role in building motivation for changing behavior toward sustainability [47].

Over half (55%) of food-related GhGs emissions are generated through storage, preparation, consumption, and transportation, while the remaining 45% is generated through food production [48]. Food businesses can instigate better practices to improve their products’ nutrition and environmental impact; within the enormous ultra-processed food groups’ items, most sports supplements are commercialized in plastic packages and contribute to environmental damage. Research about eco-friendly packages and organic or ecological ingredients could be an alternative for food businesses to lower their environmental impact.

Mindfulness training can support people to build new health routines for a range of behaviors, such as healthy eating. Geiger et al. [48] proposed that mindfulness practice is substantially mediated by healthy behavior in ecological development; it is noteworthy that health-conscious people are more likely to conserve the environment beyond direct personal health gain [47].

Moreover, a systematic literature review about mindfulness and sustainability referred to three main potential ways mindfulness could positively affect ecological behavior: reorientation toward nonmaterialistic goals, a simple lifestyle, and a cultivation of prosocial and compassionate behaviors [47]. Even in the competitive sport frame, this life profile could inspire an interesting way for athletes to cope with their daily challenges. Furthermore, an appreciation of nature is partially fueled by interaction with healthy nature, i.e., people seeking restoration in a natural setting.

Food habits are affected by multiple factors, including dietary restriction and the dictations of various religions. The factors affecting food nitrogen footprint, with possible cultural and religious associations, are classified in to three dimensions—which involve behavioral, technical, and socioeconomic influences—a high consumption of animal-based food products, low consumption of plan-based products, and an excess of food waste. Besides the enhancement of livestock production, these dimensions lead to increase of N footprint. The consumption of animal-derived food such as red meat, eggs, and dairy products accounted for nearly 72% of the global P footprint. An Indian study [49] compared India’s N and P footprints for the major religious communities (Hindus, Muslims, Christians, and Buddhists); the impact of individual choice on the N and P footprint, found by [49], suggests that a shift from a diet based on animal protein to a plant-based diet would significantly decrease individual P footprints at the Indian national level.

An economic factor, income [48], was a negative predictor of ecological behavior, showing that higher income is usually detrimental to ecological behavior.

Overall, a general positive effect of mindfulness practice is the intention to take care of one’s body [50]. A mindfulness environmental education approach, based on health and ecological behavior co-benefits, should be fruitful. In contrast, a study in the French population (*n* = 74,470) showed that higher consumption of UPFs was independently associated with having a lower income level, being male, being younger, smoking, being obese, and having a lower level of education [51]. The development of mindful nutrition and sustainable habits related to food system processing and production could also promote healthy and nutritious homemade cooking for the social gathering of family and friends for meals. Dietary guidelines using only conventional definitions of the food groups may not lead to optimal diets, since they do not consider the extent and purpose of food processing [51]. The success of this test could be related to a combination of different factors, highlighting that the energy, fluid, and macronutrient requirements were met; additionally, caffeine intake (582 mL of American coffee), equivalent to around 400–500 mg in our case (5–6 mg/kg^−1^), corresponded with the dose recommendations for this substance. Caffeine is recognized as an ergogenic aid, specifically for endurance [14,20,22], and positively affects pain reduction [52]. Endurance athletes should be mindful of nutritional strategies to mitigate muscle damage and the associated inflammation satisfying metabolic demand proteins [12]. In our case, the variated palatability of foods and fluids offered to the athlete, to avoid the lack of appetite and boredom with the flavors, could help avoid gastrointestinal complaints while reaching the protein, CHO, energy, and antioxidant recommendations.

## 5. Conclusions

The sustainable and mindful support—specifically designed for an ultra-endurance participant, mainly provided from organic, local, and seasonal plant food sources with no ultra-processed foods and no plastic waste—was suitable for this event.

Moreover, the challenge was designed as a wake-up call for athletes to rethink how they carry out their refreshments. We had a great media presence, throughout the study, especially during the challenge, where many questions were answered through Instagram concerning the composition of the beverages and the athlete’s food. This observational research may help to make ecological products visible and provide other alternative purchase options.

## Figures and Tables

**Table 1:** Types of solid food items offered during the event day.

Food Items Support Snacks Offered and Consumed
Raw and dry fruits: tangerines, kiwis, bananas, and blueberries. Vegetables: avocados and tomatoes. Fresh bread: multi cereals with seeds, and natural yeast. Aubergine marinade: eggplant, garlic, extra virgin olive oil, and several spices (homemade). Smoked cod: ready to eat. Oat Snack Bars: muesli, oat, cashew, eggs, honey, dates, seeds, and almonds (homemade). Dark chocolate (85% pure cacao).

**Table 2:** Type of drinks offered during the event day.

Drinks Support Offered and Consumed
Isotonic (6% CHO) (lemon and honey). Hypotonic (3% CHO) (hibiscus, lemon, and honey). Non-caloric drink (cinnamon, ginger, and lemon). Non-caloric plain water (choice to add lemon juice). Coffee *. Green tea *. Vegetable/poultry homemade broth *.

* warm drinks.

**Table 3:** Energy intake from different food groups.

Food Groups	Energy Intake kcal (%TEI)
**First group**: Fruits and nuts	2699 (34%)
**Second group**: Vegetables and spices	542 (7%)
**Third group**: Grains, seeds, and derivates	2218 (28%)
**Fourth group**: Beverages (cold and warm)	1727 (22%)
**Fifth group**: Animal food	292 (4%)
**Sixth Group**: Miscellaneous (Extra virgin olive oil & dark chocolate)	592 (7%)

**Table 4:** Carbohydrate intake by food groups.

Food Groups	CHO Intake (g)
**First group**: Fruits and nuts	393
**Third group**: Grains, seeds, and derivates	421
**Fourth group**: Beverages (cold and warm)	338

**Table 5:** Protein intake by food groups.

Food Groups	P Intake (g)
**First group**: Fruits and nuts	49
**Second group**: Vegetables and spices	40
**Third group**: Grains, seeds, and derivates	55
**Fifth group**: Animal food (eggs, fish, and poultry)	45

**Table 6:** Lipid intake by food groups.

Food Groups	L Intake (g)
**First group**: Fruits and nuts	77.4
**Second group**: Vegetables and spices	34.8
**Third group**: Grains, seeds, and derivates	63.5
**Fifth group**: Animal food (eggs, fish, and poultry)	11.8
**Sixth Group**: Miscellaneous (Extra virgin olive dark chocolate)	19.5

**Table 7:** Average intake per hour during the event’s six stages.

Phase (3 h Gap Each)/Meals	CHO (g/h)	P (g/h)	Fluid Intake (mL/h)
Phase1-Earlymorning/BF	71.5	9.0	643
Phase 2-Mid-morning/MMS	128.0	15.3	620
Phase 3-Afternoon/Lunch	75.2	15.7	777
Phase 4-Mid-afternoon/MAS	90.3	18.7	607
Phase 5-Evening/Dinner	77.3	8.9	443
Phase 6-Night/NS	45.1	3.0	476

**Meals**: Breakfast (BF); mid-morning snack (MMS); mid-afternoon snack (MAS); night snack (NS).

**Table 8:** Overall macronutrients related to body weight and time (h).

Average	CHO Intake (g/kg/h)	P Intake (g/kg/h)	L Intake (g/kg/h)
18 h event	0.88	0.13	0.14

**Table 9:** Time and physical intensity level during the event.

Level of Activity	Time Minutes (h)
Rest or sedentary	47 min (0.8)
Low activity	65 min (2.7)
Moderate intensity	682 min (11.1)
Hard intensity	245 min (4.1)
Very hard	0 min

## Data Availability

We have not publicly reported any scientific data of the study yet. The final degree work was defended by the student qualifying A+ (cum laude); nevertheless, these data are not published in any other publication.
